# Parentage Verification and Segregation Distortion Patterns of Microsatellite Markers in Olive Flounder (*Paralichthys olivaceus*) Full-Sib Families

**DOI:** 10.3390/ani15020176

**Published:** 2025-01-10

**Authors:** Songhyun Gwon, Eunjeong Kim, Wonse Lee, Jisung Han, Yoonkwon Nam

**Affiliations:** 1 Faculty of Fisheries Life Sciences, Pukyong National University, Nam-Gu, Busan 48513, Republic of Korea; xodus4833@naver.com (S.G.); kejung03@naver.com (E.K.); 2 All-in-One Gene Tech, Buk-Gu, Busan 46508, Republic of Korea; wslee8607@aiogenetech.com (W.L.); red5603@hanmail.net (J.H.)

**Keywords:** microsatellites, olive flounder, parentage verification, segregation distortion

## Abstract

Microsatellite markers are widely used in aquaculture for genetic analysis and breeding programs, but challenges such as segregation distortion and allelic instability can impact their effectiveness in parentage verification and inheritance studies. This study evaluated 15 microsatellite loci in seven experimental olive flounder (*Paralichthys olivaceus*) families bred through 1:1 full-sibling crosses, assessing their utility for accurate parentage and inheritance stability. Parentage assignments were conducted within an expanded pool of 647 candidate parents (including the actual 14 parents), encompassing both closely related and moderately distant individuals. Despite increased genetic diversity, assignments maintained a high accuracy rate (99.6%), demonstrating marker robustness. Differences in delta values highlighted the influence of genetic backgrounds on assignment clarity, with some groups showing reduced distinctiveness in the expanded pool. Segregation distortion was observed at certain loci, deviating from Mendelian inheritance, likely due to meiotic drive and post-zygotic selection. These findings underscore the need for empirical validation of microsatellite loci for stable inheritance and reliable parentage in diverse breeding programs, especially with genetically similar spawners.

## Simple Summary

Accurate parentage verification and inheritance stability are crucial for breeding programs in farmed fish, including olive flounder (*Paralichthys olivaceus*), a valuable species in East Asia. This study evaluated 15 microsatellite genetic markers to assess their reliability for tracking parentage and inheritance patterns in seven flounder families bred through controlled full-sibling crosses. We identified cases of segregation distortion—unexpected deviations from normal inheritance patterns—at specific genetic loci. Additionally, we explored the performance of these markers in a large candidate pool, which included closely related individuals, and achieved high accuracy in parentage assignments despite these complexities. Our findings underscore the importance of careful marker selection and validation to ensure genetic reliability and improve breeding programs for olive flounder.

## 1. Introduction

Microsatellites, short tandem repeats with 1 to 6 base pair (bp) motifs, play critical roles in chromatin organization, recombination, DNA replication, cell cycle control, and regulation of gene expression [1]. Due to their high polymorphism, essentially and majority-neutral nature, co-dominance, and suitability for systematic assays, microsatellites serve as essential genetic markers [2,3,4]. These markers have been widely applied across biological fields, particularly in aquaculture and fisheries research, where they facilitate parentage testing, population genetic studies, and marker-assisted breeding programs [1,2,3].

Despite their utility, microsatellites are inherently unstable due to their high mutation rates, often caused by DNA replication slippage or unequal recombination, which makes them susceptible to segregation distortion—deviations from expected Mendelian genotype ratios [5,6]. Here, inheritance instability refers to deviations in the expected transmission of alleles from parents to offspring, which can arise from mutations, segregation distortion, or other anomalies affecting allele inheritance patterns. Such instability and potential sources of error at genotyping microsatellites have been observed across various species, including fish, where segregation distortion poses challenges for genetic analyses, including pedigree reconstruction and genetic linkage mapping [7,8,9,10]. These deviations can lead to interpretive errors in breeding programs, underscoring the need for case-specific marker validation to mitigate biases in genetic studies.

Olive flounder (*Paralichthys olivaceus*), an economically significant species in East Asian aquaculture, has been extensively studied using microsatellite markers for applications such as genetic linkage maps and population diversity assessments [11,12,13]. However, studies have shown that the utility and discriminative power of specific microsatellite markers can vary significantly across different populations, strains, and hatchery stocks. This variability indicates that even within a single species, markers effective for one population may not apply universally, highlighting the importance of empirical validation tailored to the genetic background of each flounder group [14,15]. The issue of genetic similarity among spawners poses an additional challenge to parentage verification, as closely related or similar spawners can lead to overlapping allele profiles that reduce the clarity of parentage assignments [2,5]. This overlap complicates the ability to distinguish true parents from potential candidates, making marker validation even more critical when working with broodstock that shares a genetic background.

In recent years, efforts to develop specific-pathogen-free (SPF) olive flounder broodstock have driven the breeding of experimental families at designated farms. In such cases, accurate parentage verification using reliable molecular markers is vital to ensuring genetic traceability and supporting breeding objectives. This study aims to evaluate 15 microsatellite loci for their effectiveness in parentage verification and detection of segregation distortion within seven experimentally produced flounder families, each created through full-sibling (1:1) crosses of selected spawners. By examining the reliability and inheritance patterns of these markers, we provide insights that can enhance marker-based breeding programs and inform future research on the stability of genetic markers.

## 2. Materials and Methods

### 2.1. Experimental Fish Families

Each broodstock used to create the experimental families each year was prepared through a specific-pathogen-free (SPF) process to protect against several major bacterial and viral pathogens; these SPF-processed stocks are denoted by “S” (e.g., S7, S11). Seven full-sibling progeny groups (P1 to P7) were established through 1:1 (female/male) crosses, involving a total of 14 spawners (7 females and 7 males). The seven families used in this study represent the result of a careful selection process. From specific candidate stocks maintained under strict biosecurity control, multiple families were generated each year. However, through repeated SPF examinations of the progeny, only the families that consistently demonstrated true SPF characteristics were retained. The P1 group was created from a female and a male sourced from the 2022 S7 and S11 stocks, respectively. For the P2 group, a female from the S7 stock was crossed with a male from the SG stock, which originated from a different research facility but also underwent SPF processing. In 2023, progeny groups P3 and P4 were produced using females from the S11 and S14 stocks, respectively. In 2024, the P5 group was generated by a sibling cross (sister–brother mating) of selected individuals from the P1 group. Finally, in 2024, groups P6 and P7 were produced with females sourced from the S14 stock for both groups and males from stocks S11 and S14 for P6 and P7 groups, respectively

Gametes for the 1:1 cross were obtained from surgically removed gonads (ovaries and testes), and artificial insemination was performed using the conventional wet method. To determine the microsatellite genotype of each spawner used for artificial insemination, caudal fin clips and/or gonad biopsies were sampled in 70% ethanol and stored at 4 °C until they were used for genotype analysis. At 50~60 days post-hatch, 30~38 randomly selected progeny individuals were obtained from each family group, and caudal fin samples were individually collected, as described above, for microsatellite genotyping.

### 2.2. Genotyping of Microsatellite DNA

Total DNA was extracted from tissue biopsies (ethanol-preserved tissues) using a conventional SDS (1%)/proteinase K (100 μg/mL, final concentration) digestion method, followed by organic extraction [twice with phenol–chloroform (1:1) and once with chloroform–isoamyl alcohol (24:1)], isopropanol precipitation, ethanol (70%) washing, and RNase A treatment (100 μg/mL). The DNA was resuspended in TE buffer (10 mM Tris and 1 mM EDTA, pH 8.0), and the quantity and purify of each DNA sample were spectrophotometrically measured using a NanoDrop ND-100 (Thermo Fisher Scientific, Waltham, MA, USA). Based on absorbance readings at 260, 280, and 230 nm, the ratios higher than 1.8 and 1.9, respectively, at 260/280 nm and 260/230 nm were verified for each DNA sample. DNA integrity of DNA sample was confirmed by ethidium–bromide (Et-Br)-stained gel electrophoresis after loading 250~500 ng of each DNA sample onto a 0.7% agarose gel.

Based on published information, including genetic linkage maps of this flounder species [12,13,16,17,18], 40 reported microsatellite candidate loci were initially selected, considering factors such as polymorphic information content (PIC), motif uniformity, type of repeats, and chromosomal locations. After preliminary tests of PCR efficiency, specificity, and size polymorphism of PCR products, a final set of 15 microsatellite loci was selected based on chromatograph analysis of PCR products amplified with 5′-fluorescently labeled forward primer and non-tagged reverse primer. Information on the final set of 15 microsatellite loci and their amplification primers can be found in Table 1 and Table S1. Three multiplex amplification sets, each comprising five microsatellite loci, were performed. Fluorescent dye labeling, multiplex amplification sets, and the composition of amplification reaction are indicated in Table S2.

Approximately 100 ng of DNA was used as a template for PCR amplification (reaction volume = 15 μL). The PCR was conducted using a Sol™ h-Taq DNA polymerase (SolGent Co., Daejeon, Republic of Korea) and Veriti™ 96-Well Fast Thermal Cycler (Applied Biosystems, Waltham, MA, USA) with following thermal cycling conditions: 5 cycles at 94 °C, 58 °C, and 72 °C, each for 1 min (step 1); 5 cycles at 94 °C, 57 °C, and 72 °C, each for 1 min (step 2); and 25 cycles at 94 °C, 56 °C, and 72 °C, each for 1 min (step 3), with an initial denaturation step at 95 °C for 10 min and final extension at 65 °C for 30 min. Diluted amplification products were analyzed with a 3730XL Genetic Analyzer (Applied Biosystems) with GeneScan^TM^ 500 LIZ^TM^ Size Standard (Thermo Fisher Scientific) according to the manufacturers’ instructions. Raw data and chromatographs were examined using GeneMapper^TM^ (ver. 4.0; Applied Biosystems), and data matrices were generated in Microsoft Excel (Microsoft, Redmond, WA, USA) for further analysis.

### 2.3. Analysis of Null Allele Frequency, Parentage, and Probability of Non-Exclusion

Null allele frequencies were calculated using two approaches. First, frequencies were manually determined by directly counting null genotypes from the observed genotype matrix for each locus and family group. Second, null allele frequencies were estimated using Cervus program version 3.07 [19], which employs a likelihood-based model under Hardy–Weinberg Equilibrium (HWE) assumptions.

To evaluate the performance of the marker set, we performed two statistical tests using the Cervus software 3.07 for parentage assignment. First, we analyzed genotype data from seven full-sib families (consisting of 14 actual parents and 244 progeny, randomly mixed from family groups P1 to P7) for parent assignment. Second, we expanded the dataset by incorporating genotype data from 633 candidate spawners (272 females and 361 males from different stocks) using the same 15 loci. Each progeny was then assigned to the most likely parents (mother and father) from a pool of 647 randomly mixed candidate parents, including the original 14 actual parents.

Statistical evaluation was conducted using a likelihood-based approach in Cervus 3.07, where logarithm of the odds (LOD) scores and delta values (i.e., the difference between the LOD score of the most likely candidate parent and that of the second most likely candidate parent) were applied to estimate parentage. In the context of both-parent exclusion involving two parents and one offspring [20], the individual probability of non-exclusion for parent pair (PNE-P)—the likelihood that a randomly selected unrelated individual will not be excluded as the parent of an offspring—and the non-exclusion probability of identity (PNE-I)—the chance that two randomly selected unrelated individuals will have identical genotypes at a particular genetic marker—were calculated for each locus using Cervus 3.07. These probabilities were then combined across all loci to generate overall PNE-P and PNE-I values for the set of 15 markers used.

### 2.4. Analysis of Segregation Patterns

Assuming Mendelian co-dominant inheritance of diploid parental genotypes, we classified segregation patterns observed in the present study into four types (Type I to Type IV) as follows: Type I refers to the segregation pattern (expected ratio = 1:0) in progeny resulting from the cross of two homozygous parents, either with the same genotype (1 allele) or different genotypes (2 alleles). Type II arises from the cross between one homozygous parent and one heterozygous parent, involving either 2 or 3 alleles, with an expected segregation ratio of 1:1. Type III corresponds to the cross of two heterozygous parents with identical genotypes, producing an expected 1:2:1 ratio with 2 alleles. Type IV results from the cross between two heterozygous parents with different genotypes, yielding an expected 1:1:1:1 ratio, with either 3 or 4 alleles involved.

To analyze the segregation pattern of each microsatellite locus, Chi-square (χ^2^) goodness-of-fit tests were conducted to determine if it followed the Mendelian segregation ratio on the basis of independent assortment. Statistical significance was assessed at a level of *p* = 0.05. The *p*-value of each marker for each offspring group was transformed to its natural logarithm in order to express the degree of distortion on a continuous scale (i.e., normal distribution), and the normalized *p*-value was defined as segregation distortion value [SDV = −ln(*p*), where *p* is the *p*-value from the Chi-square test] [8]. With represented SDVs, the severity of deviation in each marker—that is, higher SDV indicating a stronger distortion (greater deviation)—was evaluated. Statistical analyses of SDVs were performed using IBM SPSS software (version 29.02), considering segregation types and the number of alleles involved. Levene’s test was used to assess the homogeneity of variances, while the Kruskal–Wallis test and one-way ANOVA were applied for group comparisons. Pairwise comparisons in the Kruskal–Wallis test were evaluated both with and without Bonferroni correction. For ANOVA, mean separation was conducted using Tukey’s HSD and Games–Howell tests.

The segregation data indicating potential distortion based on Chi-square tests and/or log-likelihood ratio tests (G-tests) were further analyzed for crosses of two heterozygous parents with same genotype (i.e., two alleles in three genotypes, *AA*, *Aa,* or *aa*, with an expected Mendelian ratio of 1:2:1). These data were subjected to the two successive Chi-square tests following previously developed formulas to identify the phase and type of selection (gametic or zygotic selection) [21]. The selection types were examined based on the balanced frequency of two alleles (first test $x_{1}^{2}$) and the distribution of different genotype frequencies (second test $x_{2}^{2}$):

$$x_{1}^{2}={\frac{\left(2np-n\right)}{n}}^{2}+\frac{{\left(2nq-n\right)}^{2}}{n}$$

$$x_{2\;}^{2}=\frac{{\left(n_{AA}-{np}^{2}\right)}^{2}}{{np}^{2}}+\frac{{\left(n_{Aa}-2npq\right)}^{2}}{2npq}+\frac{{\left(n_{aa}-{nq}^{2}\right)}^{2}}{{nq}^{2}}$$

where *p* denotes the frequency of allele *A*; *q* denotes the frequency of allele *a*; *n* denotes the total number of samples genotyped; and *n_AA_*, *n_Aa_*, and *n_aa_* represent the numbers of three genotypes, respectively.

## 3. Results

### 3.1. Microsatellite Genotype Profiles

From the manual calculation based on observed null genotypes, incidences of individuals showing null alleles varied by locus, with *POLOC03* showing the highest null rate (up to 36.8% in P1), while most other loci displayed rates from 0% to 13.2% across the seven families. Null-free genotypes were prevalent: group P3 showed no null genotype, while P1 and P2 had the highest incidence of null genotypes among progeny. Locus *POLOC15* was consistently null-free across all groups (244 individuals total), while *POLOC03* showed the highest average null incidence (8.6%). Overall, genotyping success was 98%. Conversely, the null allele frequencies inferred with Cervus software 3.07 under Hardy–Weinberg Equilibrium (HWE) assumptions ranged from −0.0954 (*POLOC14*) to 0.2688 (*POLOC12*), which was not apparently accordant with those from manual calculations (Figure S1).

### 3.2. Parent–Progeny Matching and Unexpected Offspring Genotypes

Parentage assignments in progeny groups P1, P3, P4, P5, P6, and P7—where offspring genotypes matched the parental genotypes without novel or recombinant combinations—were unequivocally confirmed across all 15 loci.

In contrast, progeny group P2 presented several individuals with unexpected genotypes at three loci: *POLOC01*, *POLOC06*, and *POLOC14*. At the *POLOC01* locus, the female and male parents had microsatellite genotypes of 69/91 and 67/91, respectively, suggesting that their offspring should have genotypes of 67/69, 67/91, 69/91, or 91/91. However, unexpectedly, three of the 38 individuals displayed the 67/67 genotype. Similarly, at the *POLOC06* locus, where the parental genotypes were 95/119 (female) and 101/119 (male), two progeny individuals exhibited genotypes of 95/133 and 95/95, which could not be easily explained by Mendelian inheritance through diploid zygote formation from haploid gametes. Furthermore, at the *POLOC14* locus, one individual out of 38 exhibited a homozygous genotype (133/133), which was inconsistent with the heterozygous parental genotypes (109/111 for the female and 113/133 for the male) (Table S3).

### 3.3. Parental Exclusion Assignment Analysis

A parental exclusion analysis involving 14 randomly mixed parents and 244 progeny from seven families showed that all offspring matched their actual mother and father without exception, regardless of familial grouping. In the expanded dataset comprising 647 candidate parents, only one individual from group P1 (out of 244 offspring) failed to match its true father. Instead, Cervus analysis identified the male parent from group P5 as the most likely candidate, even though he was not the actual father (Table S4). Excluding this single discrepancy, accurate parent–progeny relationships were confirmed for the remaining 243 offspring, resulting in a 99.6% success rate.

**Figure 1:**
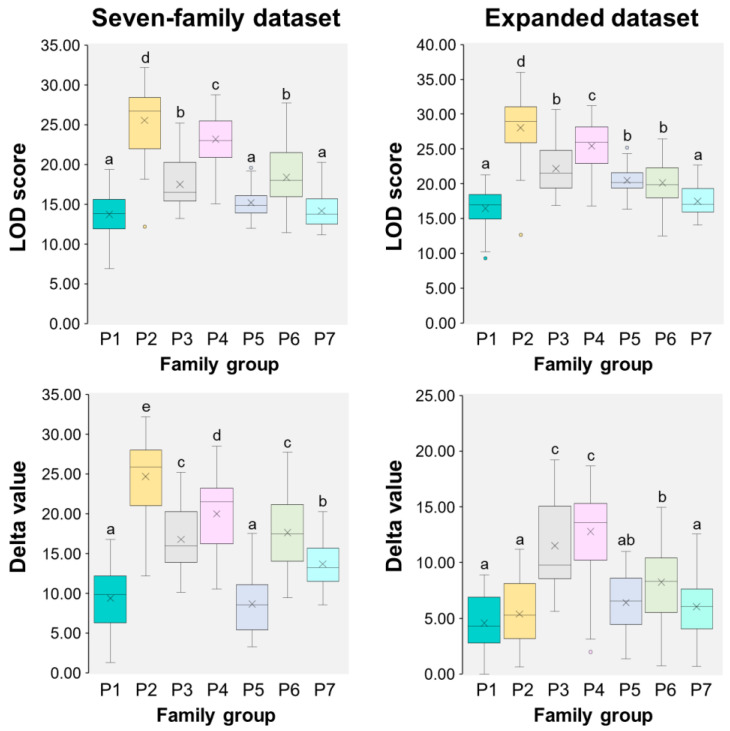
Box plots displaying LOD scores (**upper**) and delta values (**lower**) for each family group (P1–P7), assessed through Cervus-based likelihood analyses for parentage assignments within the seven-family dataset (244 progeny with 14 actual parents) and the expanded spawner pool (244 progeny with 647 candidate parents, including the 14 actual parents). Each box plot shows the median (horizontal line) and mean (cross), with whiskers representing the interquartile range. Outliers are indicated by circles. Significant differences between family groups are indicated by different letters (a–e), determined by one-way ANOVA with Tukey’s post hoc test (*p* < 0.05).

**Figure 2:**
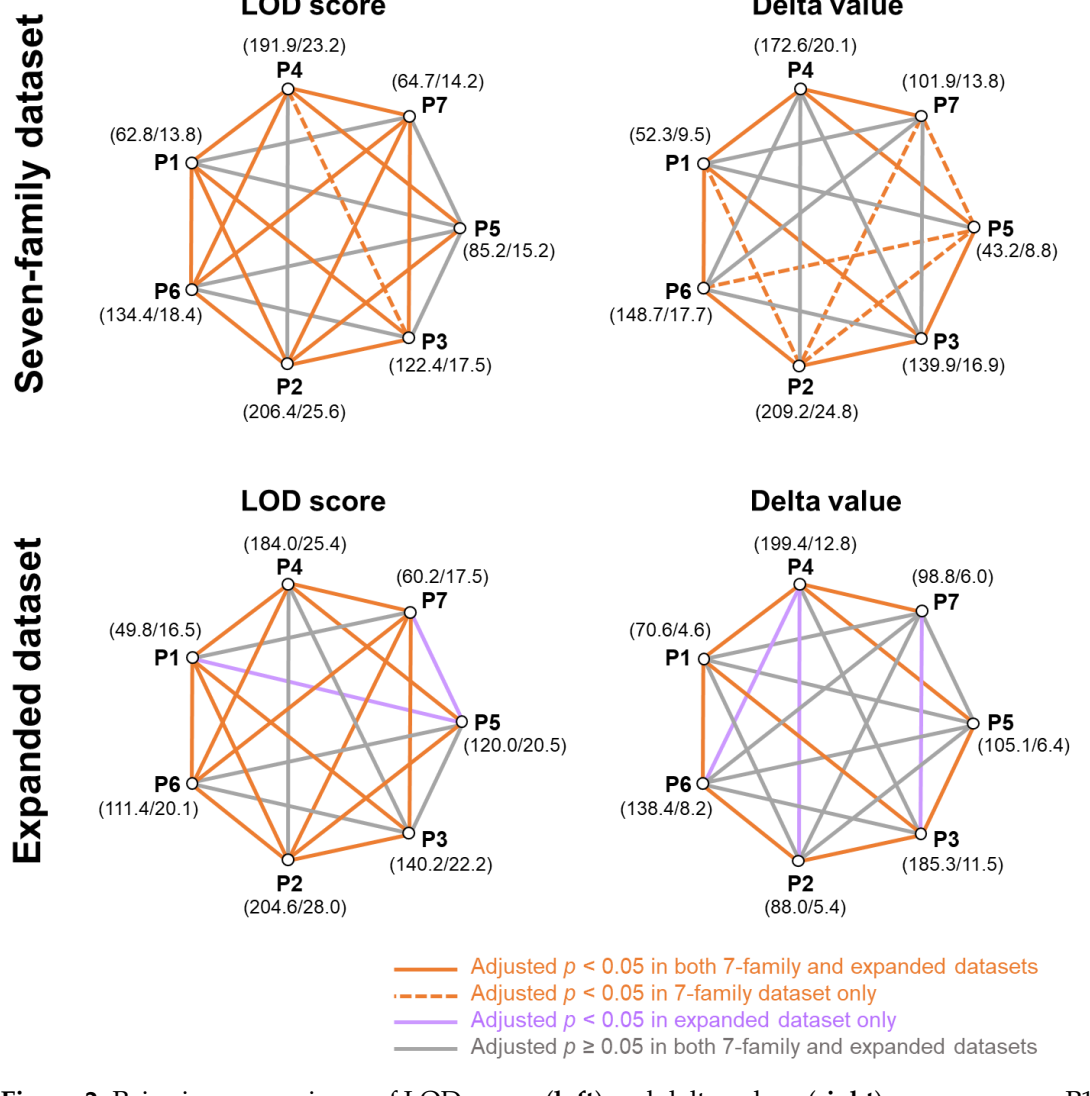
Pairwise comparisons of LOD scores (**left**) and delta values (**right**) among groups P1–P7 from Cervus-based parentage analyses within the seven-family dataset and an expanded dataset including 633 additional spawners. Statistical significance for each pairwise comparison was assessed using nonparametric Kruskal–Wallis tests with Bonferroni adjustments (adjusted *p* < 0.05). Numbers in parentheses for each group (P1–P7) represent the average rank and mean value.

Figure 1 presents the ranges of Trio LOD scores and delta values for both the seven-family and expanded datasets tested with parametric ANOVA-Tukey analysis. Nonparametric statistical evaluations based on Kruskal–Wallis pairwise comparisons and Bonferroni corrections are shown in Figure 2. In the seven-family dataset, mean Trio LOD scores from the Cervus analysis ranged from 13.84 (P1 group; lowest) to 25.57 (P2; highest). Mean delta values ranged from 8.80 (P5) to 24.78 (P2). In contrast, the expanded dataset showed a slight increase in mean Trio LOD scores, with a range from 16.47 (P1) to 28.04 (P2). However, mean delta values were generally lower across the expanded dataset, ranging from 4.57 (P1) to 12.78 (P4). Notably, P2 maintained a significantly higher mean LOD score than other groups in the expanded dataset (*p* < 0.05), but its mean delta value was low, unlike P4 and P3, which exhibited the highest mean delta values (*p* < 0.05).

**Figure 3:**
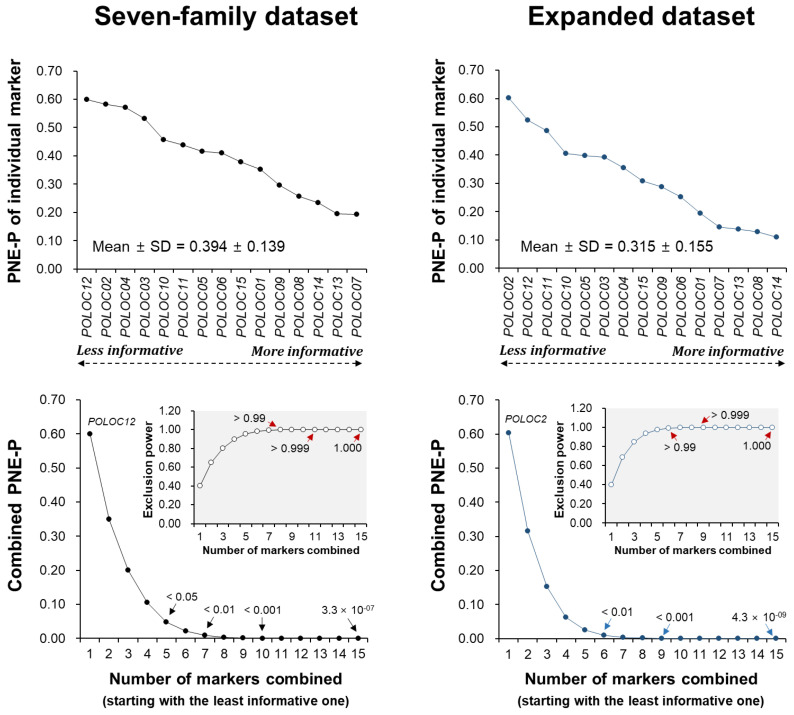
Individual (**upper**) and combined (**lower**) probabilities of non-exclusion for parent pairs (PNE-P) across 15 loci assessed using Cervus parentage analyses for both the seven-family and expanded datasets. For individual probabilities, markers are presented in ascending order of informativeness, starting from the least to the most informative. The combined probability of non-exclusion decreases as the number of markers included increases, reaching 3.3 × 10^−07^ and 4.3 × 10^−09^ when combining all 15 markers in the seven-family and expanded datasets, respectively. Insets display the exclusion power plots for added clarity.

The probabilities of non-exclusion for parental pairs (PNE-P) across the 15 loci in the seven-family dataset ranged from 0.5992 (*POLOC12*; least informative) to 0.1923 (*POLOC7*; most informative), with an average ± SD of 0.394 ± 0.139. By combining five markers (ordered by informativeness), an exclusion power exceeding 0.95 (non-exclusion probability < 0.05) was achieved, which increased to over 0.99 and 0.999 with seven and ten markers, respectively. The combined non-exclusion probability for all 15 markers was extremely low (3.20 × 10^−07^), providing near-absolute discrimination power for parental pairs. The pattern of PNE-P in the expanded dataset was similar to that of the seven-family dataset, ranging from 0.6028 (*POLOC2*) to 0.1103 (*POLOC14*), with an average ± SD of 0.315 ± 0.155 and a combined PNE-P of 4.26 × 10^−09^ (Figure 3). For identity non-exclusion probabilities (PNE-I), the combined PNE-I across all 15 markers were 6.08 × 10^−13^ and 2.22 × 10^−15^ for the seven-family and expanded datasets, respectively (Figure S2).

### 3.4. Segregation Pattern and Segregation Distortion Values

In this study, a total of 11 segregation patterns were observed, based on the co-dominant inheritance of diploid parental genotypes across seven family groups: two patterns for Type I, five for Type II, one for Type III, and three for Type IV (Table 2). The dominant segregation type varied among family groups, with Type IV being the most frequent in the majority of groups (Figure S3).

**Figure 4:**
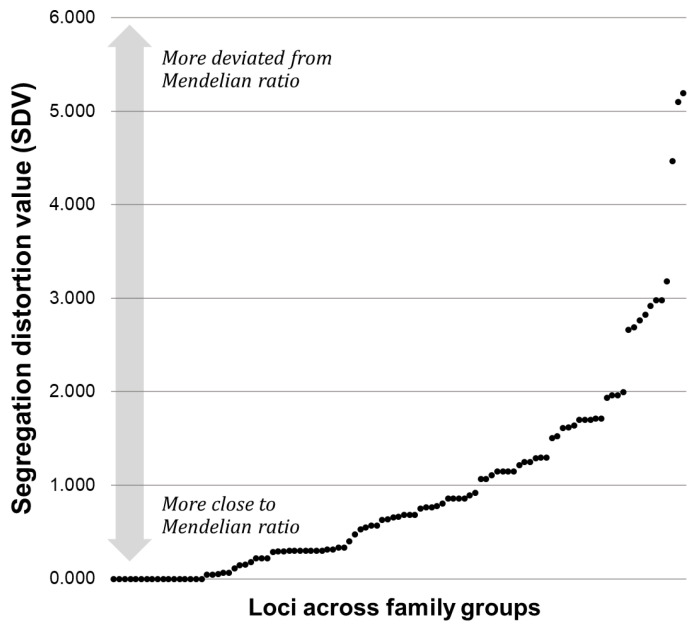
Distribution patterns of segregation distortion values (SDVs) across 15 loci in 7 family groups. SDV is calculated as the natural log transformation of Chi-square *p*-values [−ln(Chi^2^ *p*)], with higher values indicating stronger segregation distortion. The x-axis represents loci ordered sequentially by increasing SDV, with each point corresponding to a single locus.

The segregation ratio and Chi-square test p value observed at each locus across seven family groups were provided in Table S5, and the degree of segregation distortion was visualized by plotting SDVs (105 plots) (Figure 4). When arranged in ascending order, approximately 90% of the loci have SDV values below 2.0, indicating relatively low levels of segregation distortion. These loci form a continuous, tightly clustered group in the lower range of the graph. However, beyond SDV = 2.0, the pattern becomes discontinuous, with two distinct groups of loci exhibiting higher segregation distortion. The first group was observed within the SDV range of approximately 2.7 to 3.2, while the second group appeared above SDV = 4.5. These groups represent loci with significantly higher levels of segregation distortion compared to the majority of loci, which remain clustered at lower values.

**Figure 5:**
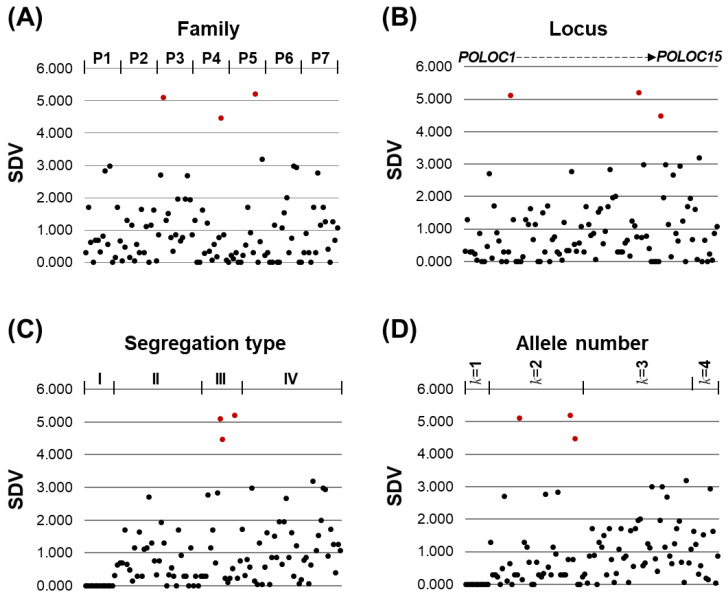
Distribution patterns of segregation distortion values (SDVs) across loci in 7 family groups. (**A**) SDV distribution by family group (P1–P7). (**B**) SDV distribution by loci, ordered sequentially from *POLOC1* to *POLOC15*. (**C**) SDV distribution by segregation types (I–IV). (**D**) SDV distribution by the number of alleles involved (*k* = 1–4). Higher SDV values indicate stronger deviations from the expected Mendelian segregation ratio. SDVs exceeding a value of 4.0, which indicate notable segregation distortion, are highlighted with red circles.

When arranged by family group (P1 to P7), several outliers with significantly higher SDVs (above 3.0) were present across multiple families but were not concentrated in any particular family group (Figure 5A). A similar pattern was observed when the data were organized by locus, with the outliers distributed across various loci without a concentration in any specific one (Figure 5B). In contrast, when classified by segregation type (I to IV), Type III exhibited the highest concentration of loci with elevated SDVs, including values greater than 3.0, indicating a higher degree of distortion in this group. Types I and II were mostly associated with low SDVs, suggesting lower levels of segregation distortion, while Type III and Type IV included loci with a broader range of SDVs, extending into the moderate-to-high distortion range (Figure 5C). Finally, when loci were grouped by the number of alleles involved (*k* = 1 to *k* = 4), loci with two alleles (*k* = 2) showed a notable concentration of higher SDVs, including the three highest SDVs. Although loci with three or four alleles (*k* = 3 and *k* = 4) exhibited greater overall segregation distortion in the lower SDV range (below 2.0), the most extreme distortions were predominantly found in two-allele loci (Figure 5D).

Taken together, both the probability of occurrence of high SDVs may be more closely related to the segregation type and number of alleles involved. In this study, only Type III loci with *k* = 2 alleles (*AA*:*Aa*:*aa*) exhibited significant distortion, while other *k* = 2 loci following different inheritance patterns (e.g., *AA*:*BB* or *AB*:*BB*) generally showed low segregation distortion values without significant deviations. However, likely due to large variations, differences in mean SDVs according to segregation types (excluding Type I) and allele numbers (excluding *k* = 1) were not clearly resolved to be statistically significant based on ANOVA and/or Kruskal–Wallis tests (Figure S4).

Segregation data showing SDVs greater than 2.0 were analyzed again with log-likelihood ratio tests (G-tests), and the seven loci representing statistical significance supported by either Chi-square or G-tests were summarized in Table 3. They originated from five family groups—P1 (*POLOC9*), P3 (*POLOC3* and *POLOC13*), P4 (*POLOC12*), P5 (*POLOC11* and *POLOC14*), and P6 (*POLOC13*)—and belonged to either Type III or Type IV segregation types. Their microsatellite genotypes can be referred to Table S5. Among these, segregation data corresponding to Type III (i.e., the expected 1:2:1 ratio) were further analyzed using two successive Chi-square tests to distinguish between meiotic (gametic, pre-fertilization) and zygotic (post-fertilization) selection. Based on the significance levels (*p* < 0.05) from both tests, the segregation distortions observed at loci *POLOC03* and *POLOC11* were likely caused by a combination of both gametic and zygotic selection. In contrast, locus *POLOC12* in group P4 indicated potential zygotic selection, as evidenced by statistical significance in the second Chi-square test but not in the first test.

## 4. Discussion

### 4.1. Genotyping Rates and Null Alleles

Discrepancies were observed at many loci between manual calculation and null allele frequency estimates generated by Cervus. These discrepancies likely arise because Cervus relies on Hardy–Weinberg Equilibrium (HWE) assumptions for null allele estimation. This approach may not be well-suited for full-sibling families, where allele distributions are shaped by Mendelian inheritance patterns and potential segregation distortion rather than random mating and population-level dynamics underpinning HWE. Notably, null alleles were prominent at locus *POLOC3* in progeny group P1, a pattern absent from other loci in P1 and from all loci in other progeny groups. The cause of the null genotype pattern at *POLOC3* in the P1 family remains an intriguing anomaly. Common sources of null alleles in microsatellite data include nucleotide divergence in primer binding sites, differential amplification favoring smaller alleles, PCR failure due to DNA template issues, and rare scenarios such as sex-linkage or aneuploidy [10,22,23,24,25].

Further characterization of the locus’s sequence could provide insights into this divergence and confirm its stability for future applications. Given that the observed null allele frequency remains below the threshold (5–10%) and is unlikely to substantially affect the accuracy of parentage analyses [24,25,26,27], *POLOC3* could be excluded from broader genetic surveys without compromising the reliability of the remaining 14 loci.

### 4.2. Occurrence of Unexpected Genotypes in P2 Progeny

Most offspring exhibited clear diploid genotypes consistent with inheritance from their respective parents across all progeny groups. However, six offspring from the P2 group showed unexpected genotypes at specific loci: *POLOC1* (three individuals), *POLOC6* (two individuals), and *POLOC14* (one individual). Among these six cases, five offspring individuals (except one individual at *POLOC6*) were homozygous at loci where both parents were heterozygous (Type IV cross). Although the precise cause of these unexpected homozygous genotypes is unclear, a potential, though untested, explanation is that a parental allele might have undergone mutation during meiotic germline transmission, resulting in a null allele that was inherited by the offspring. In this scenario, the apparent homozygous offspring may actually be heterozygotes carrying one original allele and one mutated null allele [25,28,29]. Further sequence analysis of primer-binding sites would be valuable in confirming this hypothesis. The remaining case involves an offspring with a novel heterozygous genotype (95/133) at *POLOC6*, where neither parent carried the 133-allele (female: 95/119; male: 101/119). This suggests that the new allele may have arisen from an increase in repeat length due to slipped-strand mispairing (replication slippage) rather than a null allele mutation [30,31].

### 4.3. Performance of Parentage Markers

The marker set achieved a high parentage assignment success rate of 99.6%, underscoring its robustness for parentage verification in constrained diversity contexts. One misassignment in group P1, where a progeny was incorrectly assigned to a male from P5 as its father, highlights the challenge of distinguishing closely related individuals. This case resulted from the genetic similarity between P1 and P5, as the latter was derived from a sibling cross within P1. These findings emphasize the need for additional markers or SNP-based approaches to improve discrimination power in complex family structures.

In both datasets, LOD scores increased slightly, while Delta values decreased in the expanded dataset. Higher LOD scores reflect the contribution of rare alleles from additional candidate spawners [19,32,33], whereas the reduced Delta values indicate the blurring of candidate distinctions due to shared alleles [34,35]. This pattern highlights how candidate pool composition and genetic diversity influence the clarity of parentage assignments. The distinctiveness of the P2 group, attributed to its father’s external lineage, underscores the importance of genetic backgrounds in parentage analysis [35].

While this study focused on genetically similar farm populations, future research should evaluate the marker set in more diverse genetic contexts [35,36]. Expanding the broodstock base and validating the marker system across varied populations will enhance its applicability for aquaculture breeding programs.

### 4.4. Segregation Distortion and Potential Mechanisms

Segregation distortion was observed at several loci in this study, particularly *POLOC3* (P3), *POLOC11* (P5), and *POLOC12* (P5), all displaying a Type III segregation pattern. Segregation distortion has been widely documented across fish species and is often attributed to mechanisms such as meiotic drive elements or post-zygotic selection pressures [8,37]. For example, loci *POLOC3* and *POLOC11* showed patterns indicative of both gametic (allelic) and zygotic (genotypic) selection, while *POLOC12* appeared to involve only post-zygotic selection. Meiotic drive elements, or “meiotic killers,” may explain these observations by skewing allele transmission during gametogenesis to favor their own propagation while eliminating competing alleles [9,38,39,40]. Further studies in *P. olivaceus* could investigate the presence of similar drive elements and assess their potential impacts on genetic markers used in breeding programs.

The pronounced segregation distortion at Type III loci suggests that segregation patterns, rather than allelic diversity alone, may influence inheritance stability. Specifically, loci *POLOC3*, *POLOC11*, and *POLOC12* exhibit a “recessive deleterious gene” effect, characterized by a higher-than-expected count of homozygotes (*AA*) and a lower-than-expected count of heterozygotes (*Aa*), satisfying the condition (*AA* + *aa*) > *Aa* [8]. At *POLOC3* (P3), the homozygous 280/280 genotype occurred at a notably low frequency, suggesting that the 280 allele may be deleterious. While the 276/280 (*Aa*) genotype showed moderate impact, it occurred more frequently than the 280/280 homozygotes, indicating partial tolerance when heterozygous. Conversely, the 276/276 (*AA*) genotype was observed at increased frequencies, suggesting it may be unaffected or even favored in the population. This pattern supports the hypothesis that the 280 allele exhibits a deleterious effect primarily in homozygous form, with reduced impact when heterozygous, thereby conferring a selective advantage to the 276/276 genotype.

Two loci, *POLOC13* (P6) and *POLOC14* (P5), exhibited segregation distortion in Type IV patterns, deviating from the expected 1:1:1:1 ratio for three or four alleles. At these loci, a specific allele appears to act as deleterious, reducing the proportion of genotypes carrying that allele. For example, at *POLOC13*, the frequency of allele *B*-carrying genotypes (*BC* and *BD*) decreased, while genotypes carrying allele *A* (*AC* and *AD*) increased. Similarly, at *POLOC14*, the *AB* and *BC* genotypes were less frequent, while the *AC* genotype Increased, and the *AA* genotype showed no deviation. This pattern suggests that alleles *B* at these loci may experience selective disadvantage, impacting their transmission ratios [8,41].

These results highlight the complexity of genetic transmission in *P. olivaceus*, where segregation distortion arises from multiple genetic factors, including deleterious alleles, meiotic drivers, and post-zygotic selection effects [9]. Such complexities have important implications for selective breeding programs, particularly those developing SPF stocks, as certain alleles may be preferentially transmitted under selective pressures, potentially affecting trait predictability in cultured populations [42,43]. These findings emphasize the importance of empirical validation of genetic markers before their use in breeding programs, particularly at loci prone to segregation distortion or epistatic interactions [43].

### 4.5. Limitations and Further Considerations

This study provides valuable insights into the utility of 15 microsatellite loci for parentage verification and segregation analysis in *Paralichthys olivaceus*. However, several limitations should be acknowledged. First, analyzing 30–38 individuals per family offers a foundational understanding; however, larger sample sizes could enhance the statistical power to detect subtle genetic effects and provide more robust conclusions. Second, focusing on farm broodstock populations, which may exhibit reduced genetic diversity due to selective breeding practices, limits the generalizability of our findings. Assessing these markers in more genetically diverse populations, including wild stocks, would be beneficial for broader applicability. Finally, while we observed segregation distortion at certain loci, the underlying genetic mechanisms remain unclear. Further molecular studies are necessary to elucidate these processes and their implications for breeding programs. Future research should aim to address these limitations by incorporating larger and more diverse sample populations, as well as exploring the genetic underpinnings of observed phenomena, to enhance the reliability and applicability of microsatellite markers in aquaculture genetics.

## 5. Conclusions

This study evaluated the utility of 15 microsatellite markers for parentage verification and genetic relationship assessment in *Paralichthys olivaceus*. The markers demonstrated high accuracy in parentage assignments, even within an expanded pool of closely related individuals, highlighting their robustness under challenging conditions. However, locus- and family-specific high null allele frequencies and significant segregation distortion underscore the need for thorough marker validation and careful interpretation of genetic analyses. These findings emphasize the importance of accounting for genetic backgrounds in parentage analysis and addressing potential transmission biases caused by segregation distortion. Future research should explore the genetic and epigenetic mechanisms underlying segregation distortion, including the roles of meiotic drivers and post-zygotic selection. The achievements of this study provide a strong foundation for refining genetic tools and strategies for breeding programs, enhancing their reliability and genetic stability.

## Figures and Tables

**Table 1:** List of 15 microsatellite loci and their respective amplification primers used in this study.

Locus	Repeat Motif	Forward Primer	Reverse Primer
*POLOC1* *	AC	CTCAGGCTCCACATCCCAACA	TCGTAATCAGCCCCCATCTCTGTA
*POLOC2*	TG	AAGTGAGGCTCGGGAGTTTG	GTTTCTAAACAGCTCAGGTCGTCGTT
*POLOC3*	TG	ACAGAAACACACGTTAAAGCGT	GTTTCTTCGTCTCAACAGCAGCTTGT
*POLOC4*	TATC	AGACATCTGCCCATGTTGGT	GTTTCTCACTAACCCGTAACAGGTGCT
*POLOC5*	TC	AGCTGACCTGAATGCACGAA	GTTTCTCTCCAGACAAGGTGGCTCAA
*POLOC6*	CA	ATGAAAACCACCAAGAATCCC	GGCGCATTTGGTAGTTTGTT
*POLOC7*	GT	ACTGCATGCATAACCAACAGTGTGT	GGCTGAATTATTTGGAGCAGAAGGT
*POLOC8*	AC	TCATCCCATTAAAGCATAGCG	ATCTCACAGCATCACTTGATGG
*POLOC9*	TGC	GTTGCAGTTCTTTGTGCAGACC	TCGATGTGCGCCAGAGGA
*POLOC10*	GCA	GGTACAATCCTCGGCAGTGTTC	TGAAACTCAAGCTGTTGCTGG
*POLOC11*	AGC	TGTTTGCACAGCACCATGC	TGAGGTACACACCGAGCAC
*POLOC12*	GCA	AGATTGTAGTTCGAGGTTCGTCC	TGAATCTGCTTTCCCAAGCATG
*POLOC13*	AGA	TGCTGCTCTACGCTCCAG	ACGTTGATGAAGTTCTTTCCGAGC
*POLOC14*	CA	ACAATAGGATGCAGCTGCCT	AAGCGCAAATTGTTATTCCG
*POLOC15*	CA	GAGAGACAGAAGGTCGTCAACGGTA	ACAAAGACCACGATGCAAAGTGAC

* Microsatellite markers (loci) were renumbered in this study for improved readability and flow. The original names and sources of these markers are provided in Table S1, and multiplex amplification conditions, including fluorescent dye labeling, are provided in Table S2.

**Table 2:** Segregation patterns of co-dominant microsatellite loci in offspring from seven family groups (P1–P7), classified into four types (Type I–IV) based on diploid parental genotypes.

Type	Mother	Father	Progeny				No. of Alleles	Expected Ratio	No. of Loci Following Each Segregation Type in Each Family						
									P1	P2	P3	P4	P5	P6	P7
Type I	*AA*	*AA*	*AA*				1	1:0	2	1	-	1	2	2	2
	*AA*	*BB*	*AB*				2	1:0	-	-	-	1	-	1	-
Type II	*AA*	*AB*	*AA*	*AB*			2	1:1	2	2	2	3	1	4	3
	*AB*	*AA*	*AA*	*AB*			2	1:1	1	1	-	-	2	1	2
	*AB*	*BB*	*AB*	*BB*			2	1:1	-	1	1	-	1	-	-
	*AA*	*BC*	*AB*	*AC*			3	1:1	2	1	2	1	-	-	-
	*AB*	*CC*	*AC*	*BC*			3	1:1	1	3	1	-	1	1	2
Type III	*AB*	*AB*	*AA*	*AB*	*BB*		2	1:2:1	1	-	1	1	6	1	-
Type IV	*AB*	*AC*	*AA*	*AB*	*AC*	*BC*	3	1:1:1:1	3	1	2	2	1	1	4
	*AB*	*BC*	*AB*	*AC*	*BC*	*BB*	3	1:1:1:1	1	2	5	3	1	2	2
	*AB*	*CD*	*AC*	*AD*	*BC*	*BD*	4	1:1:1:1	2	3	1	3	-	2	-

**Table 3:** Segregation distortion patterns at multiple loci across different family groups assessed by Chi-square and G-tests.

Family Group	Locus (Marker)	Segregation Type	No. of Progeny Tested	Expected Genotype and Ratio	Observed Number	SDV	G-Test *p*	$x_{1}^{2}$ *p*	$x_{2}^{2}$ *p*
P1	*POLOC9*	Type III	37	1(*AA*):2(*Aa*):1(*aa*) = 9.3:18.5:9.3	3:23:11	2.824	0.029	0.063	0.059
P3	*POLOC3*	Type III	30	1(*AA*):2(*Aa*):1(*aa*) = 7.5:15:7.5	15:11:4	5.100	0.011	0.005	0.006
P3	*POLOC13*	Type IV	30	1(*AB*):1(*AC*):1(*BC*):1(*BB*) = 7.5:7.5:7.5:7.5	2:9:7:12	2.662	0.040	N/A	0.070
P4	*POLOC12*	Type III	30	1(*AA*):2(*Aa*):1(*aa*) = 7.5:15:7.5	8:8:14	4.467	0.015	0.121	0.011
P5	*POLOC11*	Type III	36	1(*AA*):2(*Aa*):1(*aa*) = 9:18:9	4:15:17	5.194	0.008	0.002	0.006
P5	*POLOC14*	Type IV	36	1(*AA*):1(*AB*):1(*AC*):1(*BC*) = 9:9:9:9	9:6:16:5	3.179	0.053	N/A	0.042
P6	*POLOC13*	Type IV	34	1(*AC*):1(*AD*):1(*BC*):1(*BD*) = 8.5:8.5:8.5:8.5	13:12:4:5	2.921	0.046	N/A	0.054

N/A: Not available.

## Data Availability

Raw data are potentially available upon request to the authors.

## Supplementary Materials

The following supporting information can be downloaded at https://www.mdpi.com/article/10.3390/ani15020176/s1. Table S1: Original names and references (with accession codes) of microsatellite markers utilized in this study; Table S2: Fluorescent dye labeling, multiplex amplification sets, and composition of amplification reaction used for microsatellite genotyping; Table S3: Microsatellite loci genotypes in the progeny group P2 that did not match the parental genotypes, as observed in multiple individuals (n = 6); Table S4: Genotypes at 15 microsatellite loci for a P1 offspring with incorrect paternal assignment, as determined by likelihood-based parentage analysis using Cervus 3.07 software; Table S5: Chi-square test results for segregation ratios at 15 microsatellite loci across seven progeny groups. Figure S1: Normalized frequency of null-genotyped individuals by microsatellite locus and family group; Figure S2: Non-exclusion probabilities for individual identity from Cervus-based parentage analyses within the seven-family dataset and an expanded dataset including 633 additional spawners; Figure S3: Proportion (%) of each segregation type (Type I to Type IV) across progeny groups (P1 to P7); Figure S4: Statistical evaluation of segregation distortion values (SDVs; normalized chi-square *p*-values) based on segregation types (Type II, Type III, and Type IV) and the number of alleles (*k* = 2, *k* = 3, and *k* = 4).
