# Supplementary material for: Parentage Verification and Segregation Distortion Patterns of Microsatellite Markers in Olive Flounder (Paralichthys olivaceus) Full-Sib Families
Source: Animals (Basel). 2025 Jan 10;15(2):176. doi: 10.3390/ani15020176 (PMC11758611; doi:10.3390/ani15020176)
Supplement: Supplementary file 1 [file animals-15-00176-s001.zip › Animals-3323103-Suppl Data File S2(Figures S1-S4).pdf]

Supplementary Data File S2

Figure S1: Normalized frequency of null-genotyped individuals across microsatellite loci and family groups.

Figure S2: Non-exclusion probabilities for individual identity from Cervus-based parentage analyses within the seven-family dataset and an expanded dataset including 633 additional spawners.

Figure S3: Proportion (%) of each segregation type (Type-I to Type-IV) across progeny groups (P1 to P7).

Figure S4: Statistical evaluation of segregation distortion values (SDVs; normalized chi-square *p*-values) based on segregation types (Type-II, Type-III, and Type-IV) and the number of alleles ($k = 2$, $k = 3$, and $k = 4$).

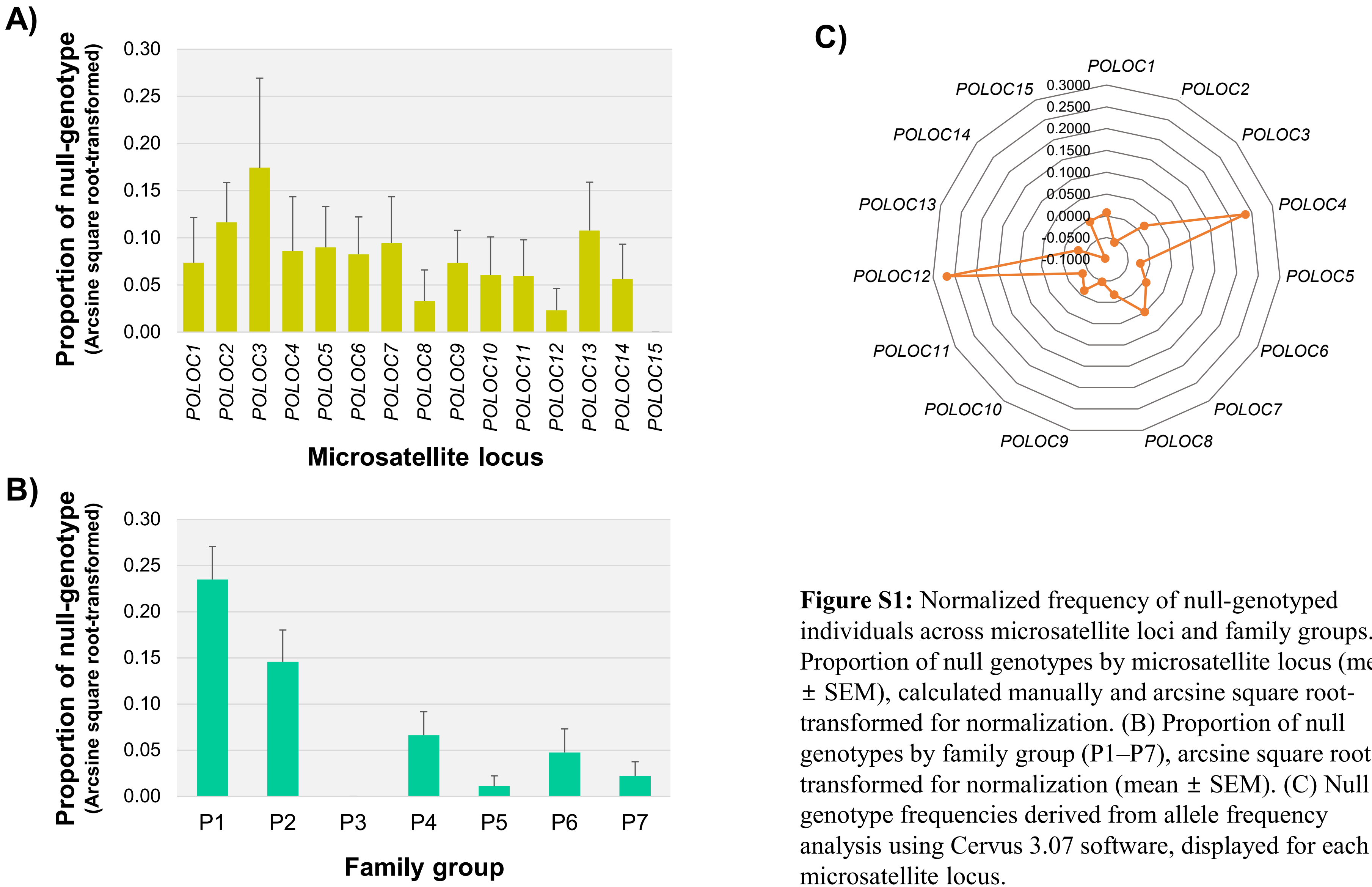

Figure S1: Normalized frequency of null-genotyped individuals across microsatellite loci and family groups. (A) Proportion of null genotypes by microsatellite locus (mean ± SEM), calculated manually and arcsine square root-transformed for normalization. (B) Proportion of null genotypes by family group (P1–P7), arcsine square root-transformed for normalization (mean ± SEM). (C) Null genotype frequencies derived from allele frequency analysis using Cervus 3.07 software, displayed for each microsatellite locus.

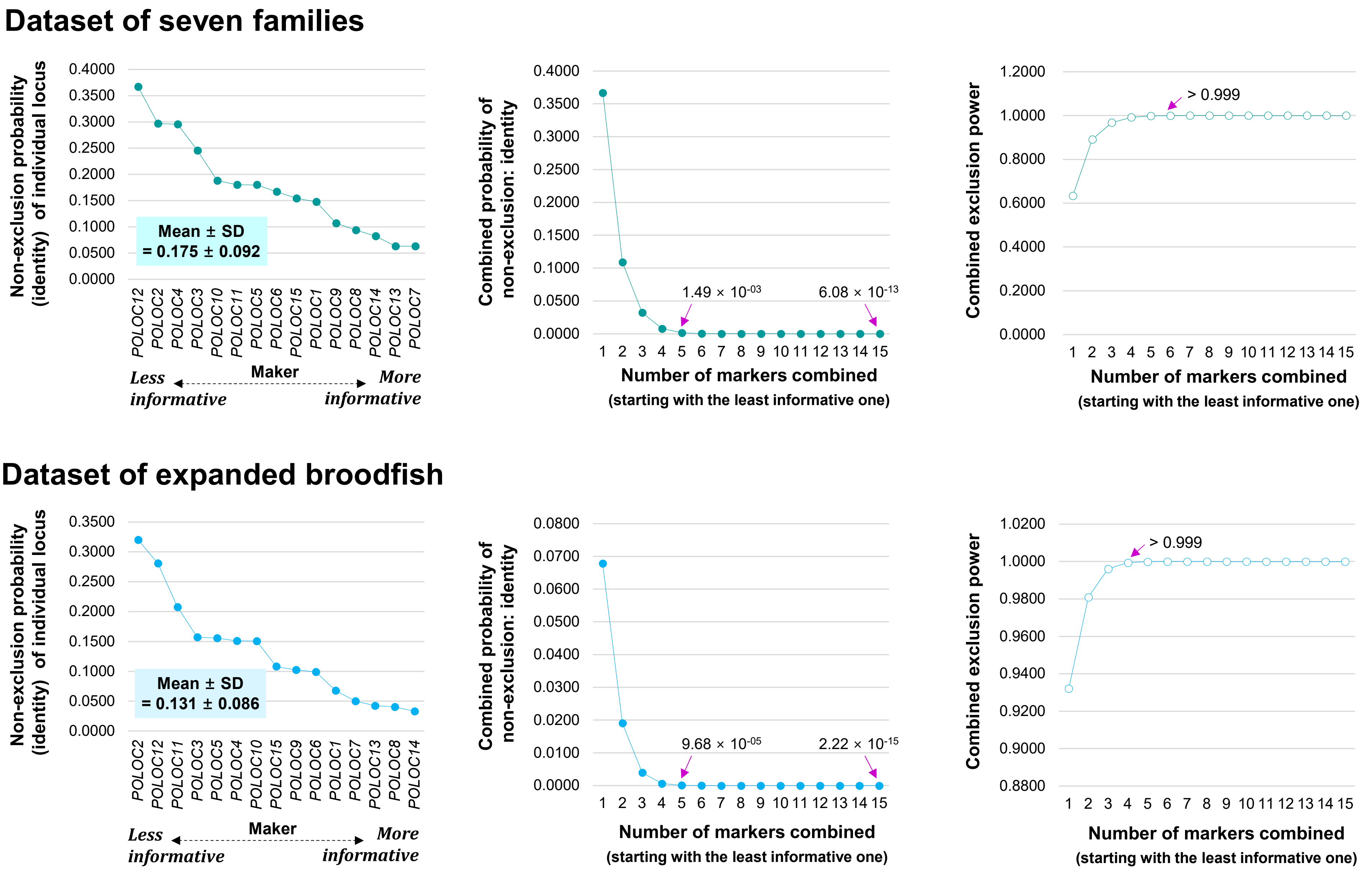

Figure S2: Non-exclusion probabilities for individual identity from Cervus-based parentage analyses within the seven-family dataset and an expanded dataset including 633 additional broodfish. The average non-exclusion probabilities per locus ranged from 0.3670 to 0.0632 in the seven-family dataset and from 0.3202 to 0.0333 in the expanded dataset. Combined non-exclusion probabilities were near zero in both datasets. Line plots for exclusion power, derived from combined non-exclusion probabilities, are also shown for each dataset.

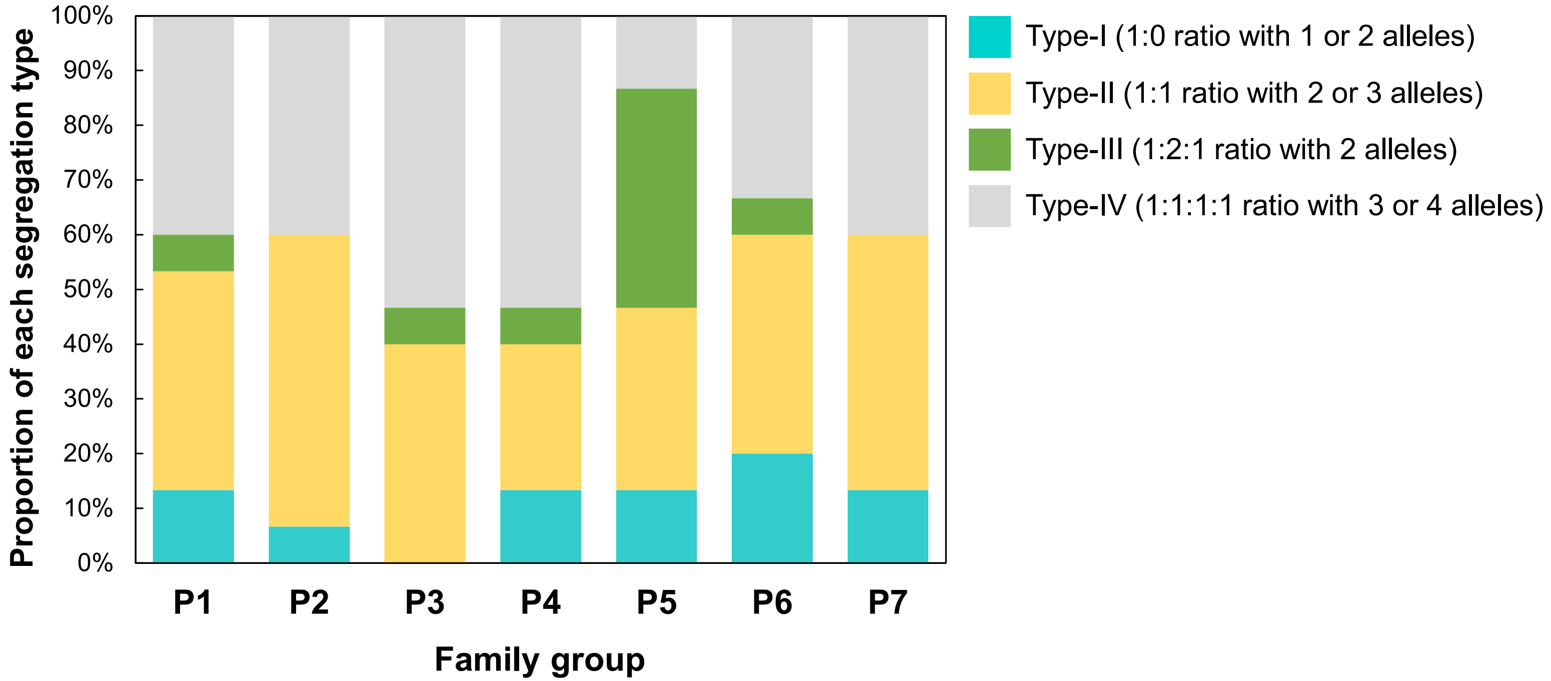

Figure S3: Proportion (%) of each segregation type (Type-I to Type-IV) across progeny groups (P1 to P7).

Tests of Homogeneity of Variances

	Levene Statistic	Significance
Based on mean	30.307	<0.001
Based on median	8.662	<0.001
Based on median and with adjusted df	8.662	0.001
Based on trimmed mean	27.943	<0.001

	Levene Statistic	Significance
Based on mean	1.878	0.159
Based on median	0.221	0.803
Based on median and with adjusted df	0.221	0.803
Based on trimmed mean	0.920	0.402

Kruskal-Wallis Tests with or without Bonferroni corrections

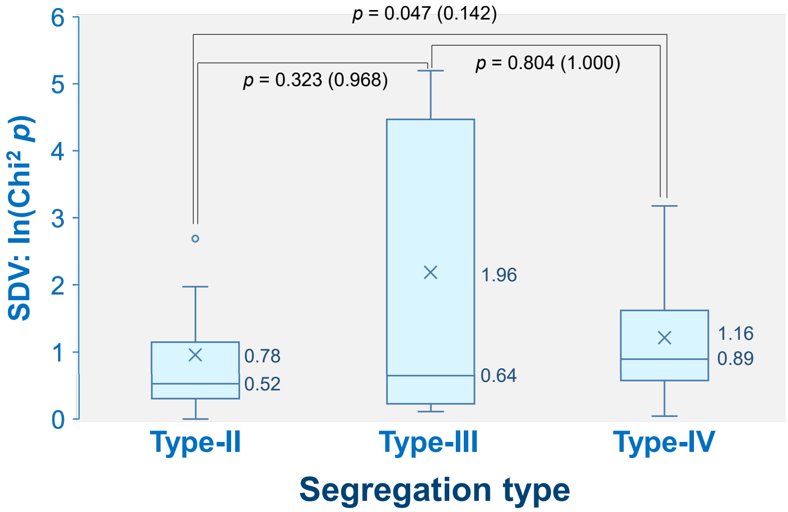

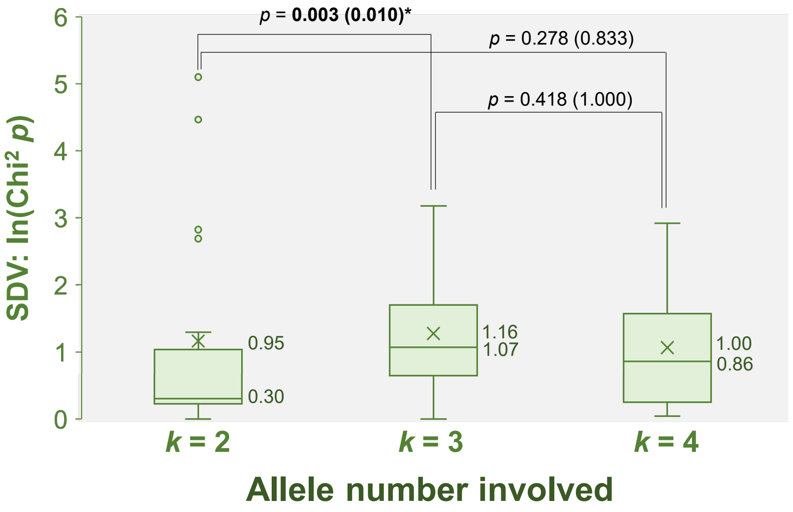

ANOVA with Tukey HSD (above diagonal) and Games-Howell (below diagonal)

	Type-II	Type-III	Type-IV
Type-II		**0.005**	0.232
Type-III	0.264		0.073
Type-IV	0.094	0.515	

	k = 2	*k* = 3	*k* = 4
k = 2		0.630	0.988
k = 3	0.662		0.897
k = 4	0.986	0.846	

Figure S4: Statistical evaluation of segregation distortion values (SDVs; normalized chi-square p-values) based on segregation types (Type-II, Type-III, and Type-IV) and the number of alleles ($k = 2$, $k = 3$, and $k = 4$). Three types of statistical analyses were conducted: Levene's test for homogeneity of variances, the Kruskal-Wallis test (nonparametric), and one-way ANOVA (parametric). For the Kruskal-Wallis test, statistical significance in each pairwise comparison was assessed with and without Bonferroni correction (adjusted *p*-values in parentheses). In each boxplot, the mean (×) and median (horizontal line) values are displayed. For ANOVA, mean separation was performed using Tukey's HSD (above the diagonal) and Games-Howell (below the diagonal) tests.
