# Supplementary material for: Parentage Verification and Segregation Distortion Patterns of Microsatellite Markers in Olive Flounder (Paralichthys olivaceus) Full-Sib Families
Source: Animals (Basel). 2025 Jan 10;15(2):176. doi: 10.3390/ani15020176 (PMC11758611; doi:10.3390/ani15020176)
Supplement: Supplementary file 1 [file animals-15-00176-s001.zip › Animals-3323103-Suppl Data File S1(Tables S1-S5).pdf]

Supplementary Data File S1

Table S1: Original names and references (with accession codes) of microsatellite markers utilized in this study.

Table S2: Fluorescent dye labeling, multiplex amplification sets, and composition of amplification reaction used for microsatellite genotyping.

Table S3: Microsatellite loci genotypes in the progeny group P2 that did not match the parental genotypes, as observed in multiple individuals (n = 6).

Table S4: Genotypes at 15 microsatellite loci for a P1 offspring individual with incorrect paternal assignment, as determined by likelihood-based parentage analysis using Cervus 3.07 software.

Table S5: Chi-square test results for segregation ratios at 15 microsatellite loci across seven progeny groups.

Supplementary Table S1. Original names and references (with accession codes) of microsatellite markers utilized in this study

Locus (marker)		Key references and accession code
**This study**	**Original name**	
POLOC1	*KOP128*	Kim et al (2013); KC947433
POLOC2	*PaOi_14**	Unpublished
POLOC3	*PaOi_31**	Unpublished
POLOC4	*PaOi_43**	Unpublished
POLOC5	*PaOi_45**	Unpublished
POLOC6	*Poli11TUF*	Castaño-Sánchez et al. (2010); AB037981
POLOC7	*Poli121TUF*	Castaño-Sánchez et al. (2010); AB037993
POLOC8	*Poli13-2TUF***	Castaño-Sánchez et al. (2010); AB459357
POLOC9	*Poli1416***	Castaño-Sánchez et al. (2010); DQ888981
POLOC10	*Poli1424***	Castaño-Sánchez et al. (2010); DQ888989
POLOC11	*Poli1427***	Castaño-Sánchez et al. (2010); DQ888992
POLOC12	*Poli1445***	Castaño-Sánchez et al. (2010); DQ889010
POLOC13	*Poli1458***	Castaño-Sánchez et al. (2010); DQ889022
POLOC14	*Poli2TUF*	Castaño-Sánchez et al. (2010); AB037978
POLOC15	*Poli9-8TUF*	Castaño-Sánchez et al. (2010); AB037989

Castaño-Sánchez et al. (2010) and Kim et al. (2013) are cited as [12] and [16] in main text, respectively.

* Primers were developed by the Korea Fisheries Resources Agency (FIRA), Busan 46041, Republic of Korea, and were approved for use in this study. ** Primer sequences (forward and/or reverse primers) were modified from the original sources to optimize PCR efficiency or facilitate multiplexing.

Supplementary Table S2. Fluorescent dye labeling, multiplex amplification sets, and composition of amplification reaction used for microsatellite genotyping. Each microsatellite locus was tagged with a specific fluorescent dye at the 5′-end of the forward primer for detection, and loci were grouped into multiplex sets (Set-A, Set-B, and Set-C) for efficient PCR amplification. Thermal cycling conditions were uniform across all sets

**Locus**	**Fluorescent dye for tagging 5′-end of forward primer**	**Multiplex set**
POLOC1	6FAM	Set-A
POLOC2	PET	Set-B
POLOC3	6FAM	Set-B
POLOC4	6FAM	Set-C
POLOC5	PET	Set-A
POLOC6	TAMRA	Set-C
POLOC7	ATTO565	Set-B
POLOC8	6FAM	Set-C
POLOC9	VIC	Set-C
POLOC10	VIC	Set-A
POLOC11	NED	Set-A
POLOC12	PET	Set-A
POLOC13	NED	Set-B
POLOC14	HEX	Set-B
POLOC15	HEX	Set-C

**Reaction compositions of multiplex PCR**											
**Multiplex Set-A**				**Multiplex Set-B**				**Multiplex Set-C**			
Component		Conc.	Vol. (μL)	Component		Conc.	Vol. (μL)	Component		Conc.	Vol. (μL)
Reaction Buffer		10X	1.50	Reaction Buffer		10X	1.50	Reaction Buffer		10X	1.50
dNTPs		10 mM	0.30	dNTP mix		10 mM	0.30	dNTP mix		10 mM	0.30
F Primer	*POLOC1*	10 μM	0.10	F primer	*POLOC7*	10 μM	0.10	F primer	*POLOC8*		0.35
	POLOC10	10 μM	0.20		*POLOC3*	10 μM	0.10		*POLOC4*		0.15
	POLOC11	10 μM	0.15		*POLOC14*	10 μM	0.15		*POLOC15*		0.15
	POLOC12	10 μM	0.25		*POLOC2*	10 μM	0.15		*POLOC9*		0.15
	POLOC5	10 μM	0.20		*POLOC13*	10 μM	0.15		*POLOC6*		0.15
R primer	*POLOC1*	10 μM	0.10	R primer	*POLOC7*	10 μM	0.10	R primer	*POLOC8*		0.35
	POLOC10	10 μM	0.20		*POLOC3*	10 μM	0.10		*POLOC4*		0.15
	POLOC11	10 μM	0.15		*POLOC14*	10 μM	0.15		*POLOC15*		0.15
	POLOC12	10 μM	0.25		*POLOC2*	10 μM	0.15		*POLOC9*		0.15
	POLOC5	10 μM	0.50		*POLOC13*	10 μM	0.15		*POLOC6*		0.15
Template DNA		100 ng/μL	2.00	Template DNA		100 ng/μL	2.00	Template DNA		100 ng/μL	2.00
Taq DNA Pol.		2.5U/	0.30	Taq DNA Pol.		2.5U/	0.30	Taq DNA Pol.		2.5U/	0.30
Distilled water		-	8.80	Distilled water		-	9.60	Distilled water		-	9.00
Total		-	15.00	Total		-	15.00	Total		-	15.00

Fluorescent dyes 6FAM, VIC, NED and PET were purchased from Thermo Fisher Scientific™, USA while HEX, TAMRA and ATTO565 were from Macrogen Co., Ltd., Korea.

Reaction Buffer (10X *Sol*™ h-Taq Reaction Buffer), dNTP mix and Taq DNA polymerase (*Sol*™ h-Taq DNA polymerase) were purchased from the manufacturer (SolGent, Co., Korea). After continuous adjustments, all primers produced similar fluorescent signals in the multiplex PCR systems.

Supplementary Table S3. Microsatellite loci genotypes in the progeny group P2 that did not match the parental genotypes, as observed in multiple individuals (n = 6).

**Fish**	**Locus**		
	POLOC1	***POLOC6***	***POLOC14***
Maternal parent	69/91	95/119	109/111
Paternal parent	67/91	101/119	113/133
Offspring individual #1	67/**67**		
Offspring individual #2			133/**133**
Offspring individual #3	67/**67**		
Offspring individual #4		95/**133**	
Offspring individual #5		95/**95**	
Offspring individual #6	67/**67**		

Supplementary Table S4. Genotypes at 15 microsatellite loci for a P1 offspring individual with incorrect paternal assignment, as determined by likelihood-based parentage analysis using Cervus 3.07 software. Shown are the genotypes of the true parents, the falsely assigned father, and the P1 offspring individual.

**Locus**	**Genotype**							
	**True mother**		**True father**		**Incorrect father**		**Offspring**	
POLOC1	81	81	71	87	71	81	71	81
POLOC2	274	276	274	278	274	276	274	274
POLOC3	276	278	276	276	276	280	276	276
POLOC4	359	359	359	359	359	359	359	359
POLOC5	375	375	373	375	375	375	373	375
POLOC6	95	95	95	103	95	103	95	103
POLOC7	119	139	137	169	137	139	119	137
POLOC8	108	122	106	108	108	108	106	108
POLOC9	262	274	262	274	262	262	262	274
POLOC10	185	191	185	194	185	185	185	185
POLOC11	213	222	219	222	213	219	213	219
POLOC12	146	146	146	146	146	146	146	146
POLOC13	346	349	337	355	349	355	346	355
POLOC14	131	133	111	111	111	133	111	131
POLOC15	132	132	136	140	132	136	132	136

Each of the 244 offspring individuals from seven family groups (P1–P7) was assigned to the most likely parents (mother and father) from a pool of 647 randomly mixed candidate parents, including 14 actual parents (seven mothers and seven fathers). Parentage was correctly assigned for 243 offspring, while one individual in group P1 was assigned to an incorrect father.

This misassignment occurred due to shared alleles between the true father and the incorrectly assigned father at multiple loci. For instance, at *POLOC*1, *POLOC4*, and *POLOC5*, the incorrect father shares identical alleles with the true father and offspring, contributing to ambiguity. At other loci (e.g., *POLOC3* and *POLOC9*), alleles differ only slightly, further influencing the likelihood scores calculated by Cervus. This highlights the role of likelihood-based thresholds and shared alleles in parentage analysis, where closely related individuals can sometimes lead to misassignments.

Supplementary Table S5. Chi-square test results for segregation ratios at 15 microsatellite loci across seven progeny groups (P1–P7; see **Tables S5-1** to **S5-7**). The expected (E) and observed (O) counts at each locus are provided, with significance assessed at p = 0.05 (NS = not significant, Sig. = significant). The total number of progeny tested at each locus excludes null individuals.

Table S5-1: Progeny group P1

Locus	Mother		Father		Progeny					Chi-square test	
					Total	Genotype		*O*	*E*	*p*	Sig.
POLOC1	81	81	71	87	35	71	81	**19**	17.5	**0.612**	NS
					35	81	87	**16**	17.5		
								**35**			
POLOC2	274	276	274	278	36	274	274	**8**	9.0	**0.180**	NS
					36	274	276	**12**	9.0		
					36	274	278	**12**	9.0		
					36	276	278	**4**	9.0		
								**36**			
POLOC3	276	278	276	276	24	276	276	**11**	12.0	**0.683**	NS
					24	276	278	**13**	12.0		
								**24**			
POLOC4	359	359	359	359	36	359	359	**36**	36	**-**	
								**36**			
POLOC5	375	375	373	375	36	373	375	**16**	18.0	**0.505**	NS
					36	375	375	**20**	18.0		
								**36**			
POLOC6	95	95	95	103	37	95	95	**20**	18.5	**0.622**	NS
					37	95	103	**17**	18.5		
								**37**			
POLOC7	119	139	137	169	34	119	137	**9**	8.5	**0.731**	NS
					34	119	169	**7**	8.5		
					34	137	139	**11**	8.5		
					34	139	169	**7**	8.5		
								**34**			
POLOC8	108	122	106	108	36	106	108	**11**	9.0	**0.446**	NS
					36	106	122	**9**	9.0		
					36	108	108	**11**	9.0		
					36	108	122	**5**	9.0		
								**36**			
POLOC9	262	274	262	274	37	262	262	**3**	9.3	**0.059**	NS
					37	262	274	**23**	18.5		
					37	274	274	**11**	9.3		

								**37**			
POLOC10	185	191	185	194	37	185	185	**9**	9.3	**0.567**	NS
					37	185	194	**10**	9.3		
					37	185	191	**6**	9.3		
					37	191	194	**12**	9.3		
								**37**			
POLOC11	213	222	219	222	36	213	219	**16**	9.0	**0.051**	NS
					36	213	222	**5**	9.0		
					36	219	222	**7**	9.0		
					36	222	222	**8**	9.0		
								**36**			
POLOC12	146	146	146	146	37	146	146	**37**	37	**-**	
								**37**			
POLOC13	346	349	337	355	35	337	346	**10**	8.8	**0.856**	NS
					35	337	349	**7**	8.8		
					35	346	355	**8**	8.8		
					35	349	355	**10**	8.8		
								**35**			
POLOC14	131	133	111	111	37	111	131	**23**	18.5	**0.139**	NS
					37	111	133	**14**	18.5		
								**37**			
POLOC15	132	132	136	140	38	132	136	**21**	19.0	**0.516**	NS
					38	132	140	**17**	19.0		
								**38**			

Table S5-2: Progeny group P2

Locus	Mother		Father		Progeny					Chi-square test	
					Total	Genotype		*O*	*E*	*p*	Sig.
POLOC1	69	91	67	91	33	67	69	**8**	8.3	**0.954**	NS
					33	67	91	**9**	8.3		
					33	69	91	**9**	8.3		
					33	91	91	**7**	8.3		
								**33**			
POLOC2	274	278	274	274	37	274	274	**17**	18.5	**0.622**	NS
					37	274	278	**20**	18.5		
								**37**			
POLOC3	276	280	276	302	34	276	276	**12**	8.5	**0.274**	NS
					34	276	280	**4**	8.5		
					34	276	302	**9**	8.5		
					34	280	302	**9**	8.5		
								**34**			
POLOC4	367	367	367	407	33	367	367	**16**	16.5	**0.862**	NS
					33	367	407	**17**	16.5		
								**33**			
POLOC5	373	375	371	371	36	371	373	**15**	18.0	**0.317**	NS
					36	371	375	**21**	18.0		
								**36**			
POLOC6	95	119	101	119	34	95	101	**9**	8.5	**0.950**	NS
					34	95	119	**7**	8.5		
					34	101	119	**9**	8.5		
					34	119	119	**9**	8.5		
								**34**			
POLOC7	119	135	127	139	37	119	127	**9**	9.3	**0.567**	NS
					37	119	139	**10**	9.3		
					37	127	135	**6**	9.3		
					37	135	139	**12**	9.3		
								**37**			
POLOC8	104	104	104	114	38	104	104	**20**	19.0	**0.746**	NS
					38	104	114	**18**	19.0		
								**38**			
POLOC9	265	274	277	277	38	265	277	**15**	19.0	**0.194**	NS
					38	274	277	**23**	19.0		
								**38**			
POLOC10	185	191	191	191	38	185	191	**18**	19.0	**0.746**	NS
					38	191	191	**20**	19.0		
								**38**			
POLOC11	219	219	222	225	38	219	222	**16**	19.0	**0.330**	NS
					38	219	225	**22**	19.0		

								**38**			
POLOC12	146	146	146	146	38	146	146	**38**	38.0	**-**	
								**38**			
POLOC13	340	346	349	349	36	340	349	**15**	18.0	**0.317**	NS
					36	346	349	**21**	18.0		
								**36**			
POLOC14	109	111	113	133	35	109	113	**4**	8.8	**0.199**	NS
					35	109	133	**9**	8.8		
					35	111	113	**9**	8.8		
					35	111	133	**13**	8.8		
								**35**			
POLOC15	140	142	136	144	38	136	140	**10**	9.5	**0.957**	NS
					38	136	142	**8**	9.5		
					38	140	144	**10**	9.5		
					38	142	144	**10**	9.5		
								**38**			

Table S5-3: Progeny group P3

Locus	Mother		Father		Progeny					Chi-square test	
					Total	Genotype		*O*	*E*	*p*	Sig.
POLOC1	69	81	71	81	30	69	71	**11**	7.5	**0.423**	NS
					30	69	81	**5**	7.5		
					30	71	81	**6**	7.5		
					30	81	81	**8**	7.5		
								**30**			
POLOC2	274	274	274	278	30	274	274	**20**	15	**0.068**	NS
					30	274	278	**10**	15		
								**30**			
POLOC3	276	280	276	280	30	276	276	**15**	7.5	**0.006**	Sig.
					30	276	280	**11**	15		
					30	280	280	**4**	7.5		
								**30**			
POLOC4	359	367	367	367	30	359	367	**18**	15	**0.273**	NS
					30	367	367	**12**	15		
								**30**			
POLOC5	373	375	371	375	30	371	373	**7**	7.5	**0.221**	NS
					30	371	375	**12**	7.5		
					30	373	375	**7**	7.5		
					30	375	375	**4**	7.5		
								**30**			
POLOC6	103	111	95	95	30	95	103	**13**	15	**0.465**	NS
					30	95	111	**17**	15		
								**30**			
POLOC7	137	137	121	161	30	121	137	**14**	15	**0.715**	NS
					30	137	161	**16**	15		
								**30**			
POLOC8	108	122	106	108	30	106	108	**8**	7.5	**0.423**	NS
					30	106	122	**5**	7.5		
					30	108	108	**6**	7.5		
					30	108	122	**11**	7.5		
								**30**			
POLOC9	274	277	259	274	30	259	274	**2**	7.5	**0.141**	NS
					30	259	277	**9**	7.5		
					30	274	274	**9**	7.5		
					30	274	277	**10**	7.5		
								**30**			
POLOC10	185	191	191	194	30	185	191	**10**	7.5	**0.519**	NS
					30	185	194	**9**	7.5		
					30	191	191	**5**	7.5		
					30	191	194	**6**	7.5		
								**30**			
POLOC11	219	219	219	222	30	219	219	**17**	15	**0.465**	NS
					30	219	222	**13**	15		

								**30**			
POLOC12	143	146	146	170	30	143	146	**6**	7.5	**0.141**	NS
					30	143	170	**13**	7.5		
					30	146	146	**6**	7.5		
					30	146	170	**5**	7.5		
								**30**			
POLOC13	346	367	349	367	30	346	349	**2**	7.5	**0.070**	NS
					30	346	367	**9**	7.5		
					30	349	367	**7**	7.5		
					30	367	367	**12**	7.5		
								**30**			
POLOC14	111	111	125	133	30	111	125	**19**	15	**0.144**	NS
					30	111	133	**11**	15		
								**30**			
POLOC15	142	144	132	136	30	132	142	**8**	7.5	**0.423**	NS
					30	132	144	**5**	7.5		
					30	136	142	**6**	7.5		
					30	136	144	**11**	7.5		
								**30**			

Table S5-4: Progeny group P4

Locus	Mother		Father		Progeny					Chi-square test	
					Total	Genotype		*O*	*E*	*p*	Sig.
POLOC1	81	81	79	81	30	79	81	**18**	15	**0.273**	NS
					30	81	81	**12**	15		
								**30**			
POLOC2	274	274	278	278	29	274	278	**29**	29	**-**	
								**29**			
POLOC3	280	280	276	280	30	276	280	**15**	15	**1.000**	NS
					30	280	280	**15**	15		
								**30**			
POLOC4	351	419	359	367	30	351	359	**9**	7.5	**0.198**	NS
					30	351	367	**7**	7.5		
					30	359	419	**11**	7.5		
					30	367	419	**3**	7.5		
								**30**			
POLOC5	367	375	371	375	30	367	371	**5**	7.5	**0.690**	NS
					30	367	375	**7**	7.5		
					30	371	375	**9**	7.5		
					30	375	375	**9**	7.5		
								**30**			
POLOC6	95	127	103	119	29	95	103	**6**	7.3	**0.297**	NS
					29	95	119	**11**	7.3		
					29	103	127	**4**	7.3		
					29	119	127	**8**	7.3		
								**29**			
POLOC7	121	121	119	121	30	119	121	**14**	15	**0.715**	NS
					30	121	121	**16**	15		
								**30**			
POLOC8	108	114	106	108	30	106	108	**7**	7.5	**0.940**	NS
					30	106	114	**7**	7.5		
					30	108	108	**7**	7.5		
					30	108	114	**9**	7.5		
								**30**			
POLOC9	268	268	262	265	29	262	268	**13**	14.5	**0.577**	NS
					29	265	268	**16**	14.5		
								**29**			
POLOC10	188	194	185	191	28	185	188	**7**	7.0	**0.836**	NS
					28	185	194	**8**	7.0		
					28	188	191	**5**	7.0		
					28	191	194	**8**	7.0		
								**28**			
POLOC11	219	222	222	225	29	219	222	**4**	7.3	**0.460**	NS
					29	219	225	**10**	7.3		

					29	222	222	**7**	7.3		
					29	222	225	**8**	7.3		
								**29**			
POLOC12	146	170	146	170	30	146	146	**8**	7.5	**0.011**	Sig.
					30	146	170	**8**	15		
					30	170	170	**14**	7.5		
								**30**			
POLOC13	340	346	340	367	30	340	340	**6**	7.5	**0.423**	NS
					30	340	346	**5**	7.5		
					30	340	367	**8**	7.5		
					30	346	367	**11**	7.5		
								**30**			
POLOC14	131	133	125	133	30	125	131	**7**	7.5	**0.940**	NS
					30	125	133	**7**	7.5		
					30	131	133	**7**	7.5		
					30	133	133	**9**	7.5		
								**30**			
POLOC15	132	132	132	132	30	132	132	**30**	30	**-**	
								**30**			

Table S5-5: Progeny group P5

Locus	Mother		Father		Progeny					Chi-square test	
					Total	Genotype		*O*	*E*	*p*	Sig.
POLOC1	71	81	71	81	36	71	71	**10**	9.0	**0.801**	NS
					36	71	81	**16**	18.0		
					36	81	81	**10**	9.0		
								**36**			
POLOC2	274	276	274	276	36	274	274	**8**	9.0	**0.895**	NS
					36	274	276	**18**	18.0		
					36	276	276	**10**	9.0		
								**36**			
POLOC3	276	276	276	280	36	276	276	**19**	18.0	**0.739**	NS
					36	276	280	**17**	18.0		
								**36**			
POLOC4	359	359	359	359	36	359	359	**36**	36	**-**	
								**36**			
POLOC5	373	375	375	375	36	373	375	**18**	18.0	**1.000**	NS
					36	375	375	**18**	18.0		
								**36**			
POLOC6	95	103	95	103	36	95	95	**8**	9.0	**0.801**	NS
					36	95	103	**20**	18.0		
					36	103	103	**8**	9.0		
								**36**			
POLOC7	137	139	137	139	36	137	137	**8**	9.0	**0.590**	NS
					36	137	139	**21**	18.0		
					36	139	139	**7**	9.0		
								**36**			
POLOC8	106	122	108	108	36	106	108	**14**	18.0	**0.182**	NS
					36	108	122	**22**	18.0		
								**36**			
POLOC9	262	274	262	262	35	262	262	**15**	17.5	**0.398**	NS
					35	262	274	**20**	17.5		
								**35**			
POLOC10	185	194	185	185	36	185	185	**19**	18.0	**0.739**	NS
					36	185	194	**17**	18.0		
								**36**			
POLOC11	213	219	213	219	36	213	213	**4**	9.0	**0.006**	Sig.
					36	213	219	**15**	18.0		

					36	219	219	**17**	9.0		
								**36**			
POLOC12	146	146	146	146	36	146	146	**36**	36	**-**	
								**36**			
POLOC13	337	349	349	355	36	337	349	**12**	9.0	**0.528**	NS
					36	337	355	**8**	9.0		
					36	349	349	**10**	9.0		
					36	349	355	**6**	9.0		
								**36**			
POLOC14	111	131	111	133	36	111	111	**9**	9.0	**0.042**	Sig.
					36	111	131	**6**	9.0		
					36	111	133	**16**	9.0		
					36	131	133	**5**	9.0		
								**36**			
POLOC15	132	136	132	136	36	132	132	**10**	9.0	**0.801**	NS
					36	132	136	**16**	18.0		
					36	136	136	**10**	9.0		
								**36**			

Table S5-6: Progeny group P6

Locus	Mother		Father		Progeny					Chi-square test	
					Total	Genotype		*O*	*E*	*p*	Sig.
POLOC1	81	81	71	81	36	71	81	**19**	18.0	**0.739**	NS
					36	81	81	**17**	18.0		
								**36**			
POLOC2	274	274	274	274	34	274	274	**34**	34	**-**	
								**34**			
POLOC3	258	276	280	280	34	258	280	**17**	17.0	**1.000**	NS
					34	276	280	**17**	17.0		
								**34**			
POLOC4	359	367	359	359	36	359	359	**21**	18.0	**0.317**	NS
					36	359	367	**15**	18.0		
								**36**			
POLOC5	371	371	371	375	36	371	371	**18**	18.0	**1.000**	NS
					36	371	375	**18**	18.0		
								**36**			
POLOC6	95	95	95	95	36	95	95	**36**	36	**-**	
								**36**			
POLOC7	119	139	119	129	36	119	119	**10**	9.0	**0.343**	NS
					36	119	129	**13**	9.0		
					36	119	139	**7**	9.0		
					36	129	139	**6**	9.0		
								**36**			
POLOC8	96	114	102	108	36	96	102	**5**	9.0	**0.217**	NS
					36	96	108	**11**	9.0		
					36	102	114	**7**	9.0		
					36	108	114	**13**	9.0		
								**36**			
POLOC9	262	274	268	274	36	262	268	**12**	9.0	**0.135**	NS
					36	262	274	**6**	9.0		
					36	268	274	**13**	9.0		
					36	274	274	**5**	9.0		
								**36**			
POLOC10	191	191	185	191	36	185	191	**19**	18.0	**0.739**	NS
					36	191	191	**17**	18.0		
								**36**			
POLOC11	219	222	219	222	36	219	219	**6**	9.0	**0.472**	NS
					36	219	222	**21**	18.0		

					36	222	222	**9**	9.0		
								**36**			
POLOC12	140	146	134	146	36	134	140	**16**	9.0	**0.051**	NS
					36	134	146	**8**	9.0		
					36	140	146	**7**	9.0		
					36	146	146	**5**	9.0		
								**36**			
POLOC13	343	349	337	346	34	337	343	**13**	8.5	**0.054**	NS
					34	337	349	**12**	8.5		
					34	343	346	**4**	8.5		
					34	346	349	**5**	8.5		
								**34**			
POLOC14	117	117	111	111	36	111	117	**36**	36	**-**	
								**36**			
POLOC15	132	132	132	136	36	132	132	**18**	18.0	**1.000**	NS
					36	132	136	**18**	18.0		
								**36**			

Table S5-7: Progeny group P7

Locus	Mother		Father		Progeny					Chi-square test	
					Total	Genotype		*O*	*E*	*p*	Sig.
POLOC1	81	81	71	81	36	71	81	**19**	18	**0.739**	NS
					36	81	81	**17**	18		
								**36**			
POLOC2	274	278	274	276	36	274	274	**9**	9	**0.409**	NS
					36	274	276	**13**	9		
					36	274	278	**8**	9		
					36	276	278	**6**	9		
								**36**			
POLOC3	280	280	276	280	36	276	280	**17**	18	**0.739**	NS
					36	280	280	**19**	18		
								**36**			
POLOC4	359	359	359	359	36	359	359	**36**	36	**-**	
								**36**			
POLOC5	367	375	367	373	35	367	367	**6**	8.75	**0.180**	NS
					35	367	373	**9**	8.75		
					35	367	375	**6**	8.75		
					35	373	375	**14**	8.75		
								**35**			
POLOC6	95	101	95	95	36	95	95	**17**	18	**0.739**	NS
					36	95	101	**19**	18		
								**36**			
POLOC7	119	137	119	119	35	119	119	**23**	17.5	**0.063**	NS
					35	119	137	**12**	17.5		
								**35**			
POLOC8	108	108	108	114	36	108	108	**15**	18	**0.317**	NS
					36	108	114	**21**	18		
								**36**			
POLOC9	262	268	274	274	36	262	274	**22**	18	**0.182**	NS
					36	268	274	**14**	18		
								**36**			
POLOC10	185	194	191	194	36	185	191	**7**	9	**0.286**	NS
					36	185	194	**14**	9		
					36	191	194	**8**	9		
					36	194	194	**7**	9		
								**36**			
POLOC11	219	222	213	222	36	213	219	**7**	9	**0.670**	NS
					36	213	222	**9**	9		

					36	219	222	**12**	9		
					36	222	222	**8**	9		
								**36**			
POLOC12	146	146	146	146	36	146	146	**36**	36	**-**	
								**36**			
POLOC13	346	367	346	349	36	346	346	**8**	9	**0.286**	NS
					36	346	349	**10**	9		
					36	346	367	**13**	9		
					36	349	367	**5**	9		
								**36**			
POLOC14	111	141	117	117	36	111	117	**20**	18	**0.505**	NS
					36	117	141	**16**	18		
								**36**			
POLOC15	132	142	132	136	36	132	132	**7**	9	**0.343**	NS
					36	132	136	**13**	9		
					36	132	142	**10**	9		
					36	136	142	**6**	9		
								**36**			
